# Evaluating the accuracy of Chat Generative Pre-trained Transformer version 4 (ChatGPT-4) responses to United States Food and Drug Administration (FDA) frequently asked questions about dental amalgam

**DOI:** 10.1186/s12903-024-04358-8

**Published:** 2024-05-24

**Authors:** Mehmet Buldur, Berkant Sezer

**Affiliations:** 1 Department of Restorative Dentistry, School of Dentistry, Çanakkale Onsekiz Mart University, Çanakkale, Türkiye; 2 Department of Pediatric Dentistry, School of Dentistry, Çanakkale Onsekiz Mart University, Çanakkale, Türkiye

**Keywords:** Artificial intelligence, Public health, Dental amalgam, Health informatics, Mercury poisoning, ChatGPT, ChatGPT-4

## Abstract

**Background:**

The use of artificial intelligence in the field of health sciences is becoming widespread. It is known that patients benefit from artificial intelligence applications on various health issues, especially after the pandemic period. One of the most important issues in this regard is the accuracy of the information provided by artificial intelligence applications.

**Objective:**

The purpose of this study was to the frequently asked questions about dental amalgam, as determined by the United States Food and Drug Administration (FDA), which is one of these information resources, to Chat Generative Pre-trained Transformer version 4 (ChatGPT-4) and to compare the content of the answers given by the application with the answers of the FDA.

**Methods:**

The questions were directed to ChatGPT-4 on May 8th and May 16th, 2023, and the responses were recorded and compared at the word and meaning levels using ChatGPT. The answers from the FDA webpage were also recorded. The responses were compared for content similarity in “Main Idea”, “Quality Analysis”, “Common Ideas”, and “Inconsistent Ideas” between ChatGPT-4’s responses and FDA’s responses.

**Results:**

ChatGPT-4 provided similar responses at one-week intervals. In comparison with FDA guidance, it provided answers with similar information content to frequently asked questions. However, although there were some similarities in the general aspects of the recommendation regarding amalgam removal in the question, the two texts are not the same, and they offered different perspectives on the replacement of fillings.

**Conclusions:**

The findings of this study indicate that ChatGPT-4, an artificial intelligence based application, encompasses current and accurate information regarding dental amalgam and its removal, providing it to individuals seeking access to such information. Nevertheless, we believe that numerous studies are required to assess the validity and reliability of ChatGPT-4 across diverse subjects.

**Supplementary Information:**

The online version contains supplementary material available at 10.1186/s12903-024-04358-8.

## Background

The potential for artificial intelligence (AI) to reduce the substantial time commitment required of dental professionals is noteworthy [1–3]. Moreover, AI holds promise in facilitating individuals to enhance their health at reduced expenses, affording personalized, anticipatory, and preventive dental care, and integrating healthcare services to cater to the needs of all individuals [2]. AI has the potential to enhance the quality of dental care by improving the precision and efficacy of diagnosis, generating superior treatment visuals, simulating results, and forecasting oral health and diseases [4, 5]. Incorporating AI systems as an additional tool for dentists can greatly enhance the provision of top-quality dental treatment. By utilizing AI, we can anticipate more accurate predictions of treatment outcomes and enhancements in the precision of diagnosis and treatment planning. Although deep learning mainly aids in dental diagnosis, AI purports to enhance accuracy and precision while also boosting dentists’ productivity [6]. AI applications have demonstrated their usefulness in various areas of endodontics, such as root fractures, periapical lesions, dental and root caries, stem cell viability, root canal system anatomy, and other related fields [5, 7].

Artificial intelligence encompasses Large Language Models (LLMs), which replicate the capability of humans to comprehend written text. Language models are developed using deep learning algorithms that employ massive quantities of textual data collected from diverse sources such as books, articles, and websites. Through the analysis of patterns and connections in the input data, language models learn to anticipate the occurrence of specific words or phrases in a particular context. The latest addition to the category of LLMs is Chat Generative Pre-trained Transformer version 4 (ChatGPT-4) [8]. Utilizing deep learning techniques, ChatGPT-4 has been trained on copious amounts of data to generate responses to user inquiries that closely resemble human-like language. This model consists of 175 billion parameters [9]. Designed to function as a dialog agent for multiple purposes, ChatGPT-4 can provide responses on a vast range of topics [10]. Possibly introducing a new era of models that can enhance the representation of the amalgamation of clinical knowledge and conversation-based interaction, ChatGPT-4 may be the pioneer [11]. As a simulated physician or patient, ChatGPT-4’s response-generating interface driven by narratives, has the potential to offer significant benefits [12]. Although demonstrating promise, these models have not yet been entirely successful in assessing clinical knowledge by means of question-answering tasks [13]. Promoting awareness and education regarding the proper utilization and concealed risks of LLMs based on AI in the medical field is crucial, according to a different study [14].

ChatGPT-4, built upon the foundation of GPT-4, has captured the attention of millions of scientists with its remarkable, human-like conversational responses [15]. In contrast to previous applications, ChatGPT-4 has developed conversational capabilities by leveraging an extensive knowledge base. This enables informative communication aimed at enriching decision-making knowledge [16]. The user-friendly feature of ChatGPT-4 streamlines the diagnostic process, bringing about a notable shift in the current landscape. Additionally, its advancements are poised to shape the digital health landscape [17–19]. Used in a wide variety of applications, including virtual assistants, chatbots, and language translation, its ability to understand and respond to natural language makes it a tool for personalized health-related applications [20]. These applications, which have the ability to provide ideas from specific disease groups to public health, from diagnosis to treatment options, are increasingly finding wider use in the field of health [20, 21].

ChatGPT-4 exhibits a broad spectrum of applications within the dentistry domain. These encompass telemedicine services in dentistry, aiding dentists in their clinical decision-making, contributing to the education of dental students, and providing support in crafting scientific articles, evaluations, and patient information [22, 23]. Customized and refined applications that utilize extensive language models have the potential to enhance the quality of dental telemedicine services [16, 24]. In the imminent future, it is anticipated that numerous language model applications will adeptly collect patient data, analyze symptoms, and generate initial diagnoses, subject to subsequent review by a human dentist [17, 25]. Particularly in underserved areas with restricted access to dental care, expansive language models may offer increased utility in dental telemedicine services.

Despite the conclusions drawn from literature reviews indicating that the presence of mercury in dental amalgam could potentially lead to adverse health effects, the current scientific evidence does not provide compelling support for a causal relationship between dental amalgam and diseases [26]. Given the inherently toxic properties of mercury in all of its forms, it is conceivable that exposure to dental amalgam could give rise to both local and systemic deleterious effects [27]. For these reasons, individuals’ demand and desire to obtain information about amalgam fillings and their removal has increased in recent years [28].

Large language models have many potential applications, ranging from streamlined dental record-keeping to the clinical decision-making process [29]. However, LLMs can provide entirely incorrect answers, generate nonsensical content, and present misleading information, causing serious concerns in critical areas such as healthcare [18]. This requires the models to be trained with dentistry teaching materials, patient records, and other relevant domain information to capture pertinent patterns, terminology, and context, ensuring increased accuracy. As a result, the models develop a profound understanding of dentistry concepts and become capable of generating contextually relevant and informative responses [22]. In order to prevent the spread of false information on the subject and for the public to receive correct information on this subject, dentistry and public health institutions provide great benefits in informing by answering the questions frequently asked by patients. There is no research in the scientific literature evaluating the answers of ChatGPT-4 to the questions frequently asked by patients about dental amalgam. The aim of the study was to ask the frequently asked questions (FAQ) about dental amalgam determined by the United States Food and Drug Administration (FDA), which is one of these information resources, to ChatGPT-4 and to compare the content of the answers given by the application with the answers of the FDA. For comparison, the brochure on the FDA website was used due to its global presence as an organization that compiles and answers FAQ related to this topic, which patients can easily access through the website.

## Methods

### Methodological framework: study setup, search engine selection, bias avoidance, and data cleaning

A new email address was created for the purpose of working to avoid any negative bias effects of search algorithms. Access to the ChatGPT-4 application was made through a search engine query using Google (Google Inc., California, United States). The ‘Continue with Google’ tab was preferred at the application login. In order to mimic a typical user experience, the most widely used search engine worldwide was chosen. Prior to the question-and-answer (Q&A) session, all search history and cookies were cleared from the computer. The ‘Clear Browsing Data’ section under the ‘Privacy and Security’ tab in the ‘Settings’ menu of the search engine was utilized. By clicking the checkboxes for ‘Cached Images and Files,’ ‘Cookies and Other Site Data,’ and ‘Browsing History,’ the ‘Time Range’ was selected to align with the timeframe of the study, and the data was cleared.

### Question selection process: source, exclusions, and inclusions

As part of the study, the first four questions out of six from the FAQ section of the FDA webpage were selected and analyzed [30]. The fifth question was excluded from the study as it inquired about material information. The sixth question was also not included in the analysis as it provided a brief explanation more in the form of guidance than information. The four questions analyzed in the study were as follows:

Q.1. What Is Dental Amalgam?

Q.2. Is Dental Amalgam Safe?

Q.3. Who Should Be Concerned About Dental Amalgam?

Q.4. Should Dental Amalgam Fillings Be Removed?

### Access and testing protocol for ChatGPT-4

ChatGPT-4 relies on a general language model to understand user questions across various topics. Therefore, answers may change over time due to technological advancements, scientific discoveries, or other factors. Hence, a weekly difference assessment was conducted to monitor potential variations. The current version of the application (GPT-4) was accessed with internet connectivity from Çanakkale/Türkiye, on May 8, 2023, at 10:00 (https://openai.com/gpt-4) [31]. The selected four questions were asked in their original language, English, without modification, and the answers were reported in English as well. The same process was repeated one week later (May 16, 2023), at the same location and time, and the responses were once again documented. The questions were asked uninterruptedly and in the same order within a single text message. The specified four questions were asked one by one and consecutively within the same conversational context (Supplementary Material 1). At the same time, the answers on the FDA web page are also recorded and listed.

### Answer comparison methodology: sources and response similarity analysis

The similarity of the responses given to the same questions with a one-week interval was also investigated by conducting a dialogue through ChatGPT-4 in the same manner. After the Q&A session held on May 8th, the responses provided by ChatGPT-4 were compared with the answers in the FDA’s informational brochure. ChatGPT-4 and FDA response texts were individually subjected to similarity checks using the ‘Doc-to-Doc Comparison’ feature in a similarity detection program (iThenticate “http://www.ithenticate.com”), and their similarities were reported. Sentiment analysis is a field of natural language processing that aims to make inferences about emotions and thoughts from texts related to certain entities and topics, and research in this field aims to investigate various aspects of one or more sentences in a text that can be associated with different emotions at different levels of detail [32]. It is important to have high accuracy in sentiment analysis in AI-based LLMs. Recent studies have reported that ChatGPT represents at least the right direction regarding specialized questions in the field of health and bio(medical) sciences [33–35]. Similarly, the increase in the effectiveness of topic modeling increases the validity of an AI-based application [36]. Since the first day ChatGPT was released, it has been reported that its effectiveness in topic modeling has increased with the updates to the program [36–38]. It has also been reported that ChatGPT has great potential in evaluating the realism of text summarization [39]. It has been shown that the text summarization proficiency of ChatGPT, which is another important parameter in AI-based applications, is quite good, and while text classification algorithms can distinguish between real and generated summaries, humans cannot distinguish between real summaries and those generated by ChatGPT [40]. Considering its reported success in all these matters, it was decided to use ChatGPT-4, the latest version of Chat-GPT, in the study.

### ChatGPT-4 response evaluation: criteria and comparison methodology

The accuracy of the responses provided by ChatGPT-4 to the specified 4 questions, along with identifying similarities and differences, has been thoroughly examined within the scope of ChatGPT-4 capabilities based on the following 4 items, and reported by the researchers;

1. **Main Idea**: Analysis for identify the central theme or message of each answers. In the answer given to a curious question on the relevant subject, this issue was taken into account as the questioner first tried to learn the main idea of the answer given [41].
2. **Quality Analysis**: The accuracy and reliability of the information obtained by the questioner basically shows the quality of the information. Especially in health sciences, accurate and real information can only be achieved with quality answers [42–44]. Evaluating each answers using relevant criteria based on the questions provided below;

### a. Relevance

a.1. Does the answer address the core of the question and avoid straying off topic?

a.2. Does the answer stay within the intended scope of the question?

### b. Accuracy

b.1. Is the information factually correct and verified where possible (or evidence-supported)?

b.1.1. Can the information be supported by relevant references?

b.2. Are there any misleading or contradictory statements?

### c. Completeness

c.1. Does the answer provide enough information to fully answer the question?

c.2. Are there any key points missing or left vague?

### d. Clarity

d.1. Is the language clear, concise, and easy to understand?

d.2. Are there any jargon or complex terms that may hinder understanding?

3. **Summary of Common Ideas**: The common ideas between the answers prepared by human and scientific authorities, and the answers ensured by AI provide an idea about the validity and reliability of AI-based applications [45]. The aim is to identify overlapping themes, concepts, or information presented in both answers.
4. **Summary of Inconsistent Ideas**: As with common ideas, inconsistent ideas also provide insight into the validity and reliability of AI-based applications [46, 47]. Areas where the answers differ in their perspectives, information, or conclusions have been investigated.

## Results

The ChatGPT-4 was used to obtain the following answer for the similarity of questions asked with a one-week interval and the similarity of the answers provided in terms of both meaning and word usage:

The two texts are quite similar in content and meaning, as both describe dental amalgam as a filling material that contains silver, tin, copper, and mercury. They both explain how the amalgam is prepared and used to fill cavities caused by tooth decay in both primary and permanent teeth. They also touch on the ongoing debate over the safety of dental amalgam due to the presence of mercury and mention the opinions of various organizations, such as the American Dental Association and the U.S. Food and Drug Administration. Overall, the two texts share similar information but present it in slightly different ways.

In the examination conducted through the word similarity program, the similarity percentages of the response texts were reported as 13%, 0%, 4%, and 9% according to the order of questions. Following this analysis, in the first Q&A session, the responses provided by ChatGPT-4 are outlined below, focusing on the main points and themes of the answers.

### ChatGPT-4 responses

#### Q.1. What is dental amalgam?

The main focus of the answer to this question is generally on dental amalgam, a commonly used filling material in dentistry. The text discusses the composition of dental amalgam, which includes silver, tin, copper, and mercury, and how it is prepared for use in tooth cavities. The text emphasizes that dental amalgam is considered safe and effective by reputable organizations such as the American Dental Association (ADA), the FDA, and the World Health Organization (WHO). Additionally, it highlights the durability and long-lasting nature of dental amalgam as a filling material.

#### Q.2. Is dental amalgam safe?

The main focus of the text is on the reliability of dental amalgam. The text acknowledges that dental amalgam contains mercury, recognizing it as a toxic substance. However, it emphasizes that the mercury in dental amalgam is in a stable compound, not easily absorbed by the body, and the amount of mercury released by dental amalgam fillings is very low and within safe limits. The text highlights the consensus of reputable organizations, such as the ADA, the FDA, and the WHO, on the safety and effectiveness of dental amalgam in dental restorations. It underscores the importance of decisions regarding dental materials being made in consultation with a dentist based on the patient’s specific needs and preferences.

#### Q.3. Who should be concerned about dental amalgam?

The text emphasizes specific groups that may be more sensitive to the potential negative effects of mercury exposure in dental amalgam. Individuals with kidney problems or allergies to metals in dental amalgam, pregnant women, and young children are mentioned as groups that could be more susceptible to potential harmful effects. The text notes that individuals with a large number of existing amalgam fillings may have a higher risk of mercury exposure over time. It highlights the importance of sharing concerns with a dentist, evaluating individual risks and benefits, and determining the best course of action based on specific needs and circumstances.

#### Q.4. Should dental amalgam fillings be removed?

The text emphasizes that the decision to remove dental amalgam fillings should be made individually, in consultation with a qualified dental professional. While acknowledging the presence of small amounts of mercury in these fillings, it suggests that the released mercury is generally considered safe. The text discourages removal solely for reducing mercury exposure, as the process itself may increase exposure. However, it acknowledges situations where removal may be recommended, such as for broken or decayed fillings or for individuals with known allergies or sensitivities. The key message is to consult with a qualified dental professional to make decisions based on individual dental and medical needs.

The comparative text analysis of the responses provided by the FDA to FAQs and the responses provided by ChatGPT-4 to the same questions was conducted by ChatGPT-4 under four main headings: ‘Main Idea,’ ‘Quality Analysis,’ ‘Common Ideas,’ and ‘Inconsistent Ideas’ (Supplementary Materials 2–5). These analyses are summerized in Tables 1, 2 and 3, and 4, respectively.

**Table 1:** The main idea analysis of ChatGPT-4 answers and FDA guidance for the same questions (Generated by ChatGPT-4).

QUESTIONS	The Central Theme of Each Text of ChatGPT-4 and FDA	
	ChatGPT-4	FDA
**Q.1** **What Is Dental Amalgam?**	The safety and effectiveness of dental amalgam as a filling material is highlighted, despite containing mercury, it is considered safe and effective by reputable organizations like the ADA, FDA, and WHO.	Provide a basic description of dental amalgam, outlining its composition and common name (“silver-filling”) due to its appearance. The mixture of metals used in dental amalgam includes mercury, silver, copper, tin, and zinc, with a specific emphasis on the significant proportion of mercury in the mixture (approximately 50% by weight).
**Q.2** **Is Dental Amalgam Safe?**	Dental amalgam’s safety, especially its mercury content, is a subject of ongoing debate and research. Organizations like the ADA, FDA, and WHO generally consider it safe, but some individuals may be sensitive to its components. It’s wise to consult a dentist to make informed decisions on filling materials.	Dental amalgam fillings can release small amounts of mercury vapor, especially during placement or removal. Ingesting small particles of amalgam is not known to pose a health risk, but inhaling mercury vapors may be risky for some. Studies have not conclusively linked amalgam to harmful health effects in the general population, though those with many fillings may have slightly higher mercury levels in their blood or urine.
**Q.3** **Who Should Be Concerned About Dental Amalgam?**	Pregnant women, young children, those with specific health conditions, and individuals with many existing amalgam fillings are more vulnerable to mercury exposure. It’s crucial to consult a dentist to determine the best approach based on individual needs.	Highlight the vulnerability of pregnant or nursing women, young children, those with allergies to mercury, and individuals with neurological or kidney issues to the adverse effects of mercury from dental amalgam. Emphasize caution in placing amalgam fillings in these groups. Mention the importance of research on mercury vapor exposure effects, especially on vulnerable populations, and signs of excessive exposure. Inform healthcare providers and patients about the risks and need for personalized decisions.
**Q.4** **Should Dental Amalgam Fillings Be Removed?**	It’s important to consult a dentist before removing dental amalgam fillings. While they release low levels of mercury, unnecessary removal solely to reduce exposure is cautioned against. Removal may be recommended for dental health reasons or due to allergies, but decisions should be based on individual needs and made in consultation with a professional.	Unless necessary for dental or medical reasons, it’s not recommended to remove intact dental amalgam fillings. Highlight risks like tooth damage and temporary exposure to more mercury vapor. Even for higher-risk groups like pregnant women and children, removal should be considered only when medically necessary. Discuss with a dentist or physician, especially for those with health conditions or sensitivities.

**Table 2:** The summary of Quality Analysis ChatGPT-4 answers and FDA guidance for the same questions (Generated by ChatGPT-4).

		Answer 1		Answer 2		Answer 3		Answer 4	
		ChatGPT-4	FDA	ChatGPT-4	FDA	ChatGPT-4	FDA	ChatGPT-4	FDA
**Relevance**	***a.1.*** Does the answer address the core of the question and avoid straying off topic?	Yes	Yes	Yes	Yes	Yes	Yes	Yes	Yes
	***a.2.*** Does the answer stay within the intended scope of the question?	Yes	Yes	Yes	Yes	Yes	Yes	Yes	Yes
**Accuracy**	***b.1.*** Is the information factually correct and verified where possible (or evidence-supported)?	Yes	Yes	Yes	No	Yes	Yes	Yes	Yes
	***b.1.1.*** Can the information be supported by relevant references?	Yes	No	Yes	Yes	Yes	Yes	Yes	Yes
	***b.2.*** Are there any misleading or contradictory statements?	No	No	No	No	No	No	No	No
**Completeness**	***c.1.*** Does the answer provide enough information to fully answer the question?	Yes	Yes	Yes	Yes	Yes	Yes	Yes	Yes
	***c.2.*** Are there any key points missing or left vague?	No	No	No	No	Yes	Yes	Yes	Yes
**Clarity**	***d.1.*** Is the language clear, concise, and easy to understand?	Yes	Yes	Yes	Yes	Yes	Yes	Yes	Yes
	***d.2.*** Are there any jargon or complex terms that may hinder understanding?	No	No	No	No	No	No	Yes	No

**Table 3:** The summary of common themes, concepts, or information in responses from ChatGPT-4 and FDA guidance to the same questions (Generated by ChatGPT-4).

QUESTIONS	Common Ideas of ChatGPT-4 and FDA
**Q.1.** **What Is Dental Amalgam?**	1. Both answers discuss dental amalgam, a common filling material used in dentistry. 2. They mention the composition of dental amalgam, which includes mercury, silver, tin, and copper. 3. Both describe the process of preparing and placing dental amalgam into tooth cavities. 4. They touch upon the debate surrounding the safety and effectiveness of dental amalgam. 5. ChatGPT-4 highlights endorsements from organizations like the ADA, FDA, and WHO. 6. FDA emphasizes the mercury content in dental amalgam.
**Q.2.** **Is Dental Amalgam Safe?**	1. Both answers discuss safety concerns about dental amalgam, especially regarding mercury and its potential release in vapor form. 2. They mention assessments by organizations like the ADA, FDA, and WHO regarding dental amalgam safety. 3. Both acknowledge the possibility of allergic reactions or sensitivities to the metals in dental amalgam. 4. They mention the availability of alternative filling materials. 5. Both emphasize the importance of informed decision-making with a dentist based on individual needs and preferences.
**Q.3.** **Who Should Be Concerned About Dental Amalgam?**	1. Both answers discuss the risks of mercury exposure from dental amalgam, especially for vulnerable groups like pregnant women, young children, nursing mothers, and individuals with specific health conditions. 2. They emphasize the importance of discussing concerns with a qualified dental professional. 3. Both acknowledge the limited data on the health effects of mercury exposure from dental amalgam in certain populations, highlighting the need for further research.
**Q.4.** **Should Dental Amalgam Fillings Be Removed?**	1. Both emphasize the importance of individualized decision-making with a dental professional regarding the removal of dental amalgam fillings. 2. They highlight that intact amalgam fillings, especially those without decay beneath them, generally do not need to be removed solely to reduce mercury exposure. 3. Both caution against unnecessary removal, as it may lead to loss of healthy tooth structure and temporary increase in mercury vapor exposure. 4. They stress the need to consider medical necessity and specific health conditions when deciding on removal and replacement of amalgam fillings. 5. Overall, they advocate for a careful assessment of individual circumstances and dental health needs before deciding on dental amalgam removal.

**Table 4:** The summary of areas where the answers differ in their perspectives, information, or conclusions (Generated by ChatGPT-4).

		ChatGPT-4	FDA
**Q.1** **What Is Dental Amalgam?**	a. ***Composition***	ChatGPT-4 lists metals in dental amalgam without specifying proportions.	FDA specifies that dental amalgam is around 50% mercury by weight.
	b. ***Perspective on Safety***	ChatGPT-4 reassures about the safety of dental amalgam, citing support from reputable organizations.	FDA indirectly raises concerns about mercury content, potentially implying health risks.
	c. ***Use of Terminology***	ChatGPT-4 calls dental amalgam a “commonly used filling material.”	FDA mentions dental amalgam as “sometimes called a ‘silver-filling’ due to appearance,” offering an alternative term.
**Q.2** **Is Dental Amalgam Safe?**	a. ***Perspective on Safety***	ChatGPT-4 acknowledges mercury’s presence in dental amalgam, presenting it as a toxic substance but emphasizes its stable compound form and minimal release, backed by reputable organizations.	The FDA also recognizes mercury vapor release during dental procedures, particularly placement or removal of fillings, highlighting inhalation risks. It suggests no health risks from ingesting amalgam particles but potential harm from inhaling mercury vapor.
	b. ***Presentation of Information***	ChatGPT-4 offers a comprehensive safety overview, discussing reassurances from organizations like ADA, FDA, WHO, and potential concerns about metal allergies.	The FDA focuses narrowly on mercury vapor release risks, avoiding broader safety discussions.
	c. ***Conclusion on Harmful Effects***	ChatGPT-4 concludes no evidence of harmful effects from dental amalgam based on organizational reviews.	The FDA suggests inconclusive evidence on harmful effects, implying uncertainty rather than outright affirming safety.
**Q.3** **Who Should Be Concerned About Dental Amalgam?**	a. ***Coverage of Susceptible Groups***	ChatGPT-4 identifies pregnant women, young children, individuals with certain health conditions, and those with numerous amalgam fillings as susceptible to mercury exposure.	The FDA expands on susceptible groups, including pregnant women, nursing mothers, young children, individuals allergic to mercury, and those with neurological or kidney issues. It provides more detailed discussions on potential effects.
	b. ***Depth of Information***	ChatGPT-4 provides a general overview without specific considerations or effects on infants and children.	The FDA offers in-depth information on each susceptible group, including considerations for pregnant women, nursing mothers, and children. It discusses potential effects on infants and children’s neurological systems due to mercury vapor exposure.
	c. ***Conclusions on Health Effects***	ChatGPT-4 stresses the importance of discussing concerns with dental professionals but refrains from definitive conclusions on health effects.	The FDA acknowledges limited clinical data but suggests signs of excessive mercury exposure. It advocates a cautious approach for susceptible groups, emphasizing the need for further research and individual sensitivity consideration.
**Q.4** **Should Dental Amalgam Fillings Be Removed?**	a. ***Conditions for Removal***	ChatGPT-4 emphasizes that the decision to remove dental amalgam fillings should be made on an individual basis, considering factors such as dental and medical needs, as well as circumstances. It mentions situations such as broken, worn, or decayed fillings, or allergies/sensitivities to metals, where removal may be recommended.	FDA advises against the removal of intact amalgam fillings, particularly if the filling is in good condition and there is no decay beneath it. It suggests that removal may result in unnecessary loss of healthy tooth structure and temporary exposure to mercury vapor. It only recommends removal if it is considered medically necessary by a healthcare professional.
	b. ***Perspective on Risk***	ChatGPT-4 acknowledges the potential risk of increased exposure to mercury vapor and particles during the removal process. However, it suggests that removal may be warranted in certain situations, such as allergies or sensitivities to metals.	FDA highlights the potential risks associated with removal, including loss of healthy tooth structure and temporary exposure to mercury vapor. It strongly advises against removal unless it is deemed medically necessary.
	c. ***Recommendations for Decision-Making***	ChatGPT-4 emphasizes the importance of consulting with a qualified dental professional to make informed decisions about removal, based on individual circumstances and needs.	FDA underscores the need for discussions with a dentist or physician, particularly for individuals with health conditions such as allergies, sensitivities, or neurological/kidney disease.

## Discussion

Considering the effects of LLMs-based AI applications, which have been increasingly used in many fields, including the field of health, in recent years, the main purpose of this study was to investigate the accuracy and effectiveness of ChatGPT-4’s answers to FAQ about dental amalgam by comparing them with the answers given by the FDA. After the evaluation, it was observed that although ChatGPT-4’s answers to FAQ obtained from the FDA’s website regarding dental amalgam, its effects, and removal were mostly different in form, they provided accurate and effective information in content. The importance of patients accessing accurate and reliable information about their health has once again emerged as face-to-face communication decreased during the pandemic period [48]. Considering the sociological effects of developing technologies, individuals’ desire to access information on health-related issues through AI-based applications has also increased [49]. This has paved the way for the development of LLMs-based applications such as ChatGPT-4, beyond searches via the standard and traditional search engine. Considered in this context, taking into account the limitations and reservations, it is important for these applications to provide accurate and reliable information to users who want to access information, in terms of individual and social health in the field of health [7]. In this study, it has been shown that ChatGPT-4 provides accurate and reliable answers about dental amalgam, its effects, and removal.

Considering the increasing use in the field of healthcare, ChatGPT-based scientific publications in this context in recent years have reported that the application is a potentially valuable tool for both healthcare providers and patients in facilitating the diagnosis, treatment and prevention of various diseases [7, 50–52]. Although it has been launched very recently, it is known to provide very high rates of accurate information on topics in medical and dental contexts [7, 52]. And as natural language processing technology continues to advance, ChatGPT’s accuracy will likely increase, expanding its potential applications in healthcare [36]. In a study comparing the answers of experts and ChatGPT on endodontic questions, it was reported that although ChatGPT was not yet sufficient for clinical decision-making, it could potentially be used with the development of the application [53]. In another study, ChatGPT’s answers to 30 questions about tooth-supported fixed prostheses and removable dental prostheses were evaluated by different experts and it was reported that ChatGPT could not replace a dentist [54]. Vaira et al. [55] evaluated ChatGPT’s capacity to answer questions and solve clinical scenarios related to head and neck surgery. The researchers noted that the results generally showed a good level of accuracy in the AI’s answers, and its ability to solve complex clinical scenarios was promising [55]. Hatia et al. [56], in their study in which they investigated the accuracy and completeness of ChatGPT’s answers on interseptive orthodontics and clinical scenarios, concluded that the accuracy of the answers given by AI was highly accurate and complete, although not 100%. In another study where the answers given by the application to FAQ about orthodontics were evaluated, it was reported that the answers were not based on any scientific article and were reliable at a moderate level [57]. Mago et al. [58] reported 100% accuracy in identifying radiographic landmarks with ChatGPT for questions regarding anatomical accidents, oral and maxillofacial pathologies, and radiographic features of oral and maxillofacial pathologies, respectively. Since not long has passed since its launch and it is a developing technology, there are not many studies in the scientific literature evaluating ChatGPT’s answers to questions about dental issues and clinical scenarios. As stated, the results of the few available studies vary. While some researchers found ChatGPT’s answers highly adequate, reliable, and accurate, others found them inadequate or moderately adequate.

Dental amalgam, which has been preferred in restorative dentistry for many years due to its longevity and durability, has been the subject of many studies in recent years due to the possible allergic and toxic effects of the mercury it contains. Due to the increase in individual and communal sensitivity to mercury in recent years, many patients apply to clinics to have their amalgam fillings removed [59]. Patients stated that headaches, fatigue, dizziness, memory and concentration impairment, anxiety, depression, irritability, and various musculoskeletal and gastrointestinal symptoms, which they often thought to be caused by dental amalgams, decreased when they removed their amalgam fillings [28, 59].

The gradual reduction of dental amalgam use as part of the Minimata Mercury Convention is one of the five key projects of the WHO’s Oral Health Work Plan 2018–2020. The WHO aimed to provide assistance to low-income countries to accelerate their phasing out of amalgam use in the timeline from April 2018 to December 2023 [60]. Although the use of dental amalgam has decreased in many countries in order to support the use of these techniques and materials under appropriate conditions in the presence of better and more reliable restorative techniques and materials and to minimize the harmful environmental effects of mercury, the International Association of Dental Research (IADR) confirms the safety of dental amalgam for the general population without allergy to amalgam components or severe renal diseases.

The European Union Scientific Committee on Emerging and Newly Identified Health Risks (SCENIHR) and the US Agency for Toxic Substances and Disease Registry (ATSDR) reported that amalgam fillings should not be removed except for allergic reactions [61–63]. In addition, it has been shown that there is no significant decrease in blood mercury levels after many years in patients who have had amalgam fillings removed [63]. As one study stated in their qualitative data-based studies, it was concluded that it remains unclear how important it is to replace dental amalgam fillings in terms of improving health complaints [64]. In light of the developments in dental amalgam that have come to the fore in recent years, it is important for patients to have accurate information about this subject, and the accuracy of ChatGPT-4’s answers to questions about this subject is also important.

In addition to its positive aspects such as the convenience it offers in healthcare services, providing an alternative to patient-physician communication, and patients’ easy access to information in difficult conditions such as pandemics, there are also some important difficulties in using ChatGPT-4 in healthcare services. Although the results of our study show that ChatGPT-4’s responses on dental amalgam are consistent with the FDA’s responses, the most significant potential issue with relying on AI technology for healthcare decision-making is the ethical implications. Patients may have difficulty trusting an AI-based technology, and there are concerns about bias in the data used to train the system. In addition, the findings obtained in these AI-based applications as a result of data obtained from patients raise concerns about the security and violation of patient data [20, 21]. The training data of ChatGPT-4 may include questions and answers from the examined FDA website, raising concerns that its responses might merely be rephrased versions of the website’s content. Additionally, it’s crucial to emphasize that ChatGPT lacks the ability for independent scientific reasoning; instead, it can only produce responses relying on recognized patterns and structures within the text it has been trained on [65, 66]. However, the authors have used a plagiarism detection program to confirm the originality of ChatGPT-4’s responses when compared with the information on the website, alleviating this concern.

Considering the importance of oral and dental health, the high incidence of oral and dental diseases, the existence of different opinions on dental materials and methods, and the developments in technology, the efforts of dentists to provide oral and dental health for people are very important [67]. In this context, patients should be informed accurately. With the effects of the pandemic period, many of the patients are researching many subjects on web-based platforms instead of consulting clinics where the risk of transmission is high [68]. However, the validity and reliability of the information on web-based sites and applications are controversial. Studies on many different subjects have evaluated the accuracy of the information on different platforms, and it has been concluded that the Internet, which is like a vast ocean, will not replace the patient-physician relationship in the field of health [69]. However, the dramatic increase in the use of the Internet and AI in the developing world shows that people’s applications to the Internet for health, as well as for many other subjects, are increasing [70–72]. For this reason, it is of great importance to present accurate and reliable information to individuals who need access to it.

In the Bloom classification, which is a classification of question types based on cognitive and hierarchical criteria based on medical education, there are question types on a scale ranging from lower-order to higher-order [73]. While lower-order cognitive skills are such as recall and comprehension, higher-order thinking skills include application, analysis, evaluation, and creation [74]. While LLMs-based applications can adequately respond to lower-order skills, they are not as successful in higher-order skills [56]. For this reason, the Bloom classification of the questions asked to AI applications in studies is also important and should also be considered as a limitation [74]. The fact that all four questions obtained from the FDA’s website in our study were at a lower-order level may explain the accuracy of ChatGPT-4’s answers to questions about dental amalgam and its effects. It is of great importance to evaluate the answers by asking higher-order level complex questions to these LLMs-based applications in future studies. As a matter of fact, the fact that the answers are not sufficient in some studies proves this. We believe that, in the context of dentistry, studies on issues that frequently worry patients and cause information pollution in society will contribute to the scientific literature and individual and social health.

Today, when the use of AI-based applications is increasing, it is known that ChatGPT-4 is used by physicians and patients to access accurate information. Since it is a fairly new application, it is a subject on which scientific research continues. In this context, aspects such as being the first study to evaluate the answers of AI on dental amalgam and being based on the questions on the website of the FDA, one of the institutions with the highest international recognition, validity and reliability regarding FAQ, constitute the strengths of our study. On the other hand, the fact that a limited number of questions were evaluated and therefore statistical analysis was not possible constitute the main limitations of our study. ChatGPT-4, like many other large language models, faces several significant challenges that impact its reliability and effectiveness. Firstly, its limited understanding of contextual meaning hinders its ability to conduct critical analysis and verify the accuracy of information. While adept at identifying patterns, it lacks the capacity for deep comprehension [75]. Secondly, the model inherits biases and errors present in its training data, which can manifest in its generated text, potentially leading to misleading or inaccurate results [76]. Lastly, the echo chamber effect poses a risk, as ChatGPT-4 may inadvertently perpetuate existing biases by recycling and reframing previously generated information without undergoing rigorous critical evaluation [77]. Moreover, since ChatGPT-4 has limited knowledge of scientific developments that occurred after January 2022, it can provide incomplete and misleading information in response to current questions, which is another significant limitation. These challenges underscore the importance of ongoing research and development to address the limitations of large language models like ChatGPT-4.

## Conclusions

The findings of this study show that the answers given by ChatGPT-4, which is seen as a potential information provider and is increasingly used in today’s world where technology is constantly developing, about dental amalgam, its effects, and removal, are compatible in content with the answers given by the FDA to the same questions.

### Electronic supplementary material

Below is the link to the electronic supplementary material.

Supplementary Material 1

Supplementary Material 2

Supplementary Material 3

Supplementary Material 4

Supplementary Material 5

## Data Availability

The datasets underpinning the outcomes of this study can be obtained from the corresponding author, B.S., upon a reasonable and substantiated request.
