# Supplementary material for: Evaluating the accuracy of Chat Generative Pre-trained Transformer version 4 (ChatGPT-4) responses to United States Food and Drug Administration (FDA) frequently asked questions about dental amalgam
Source: BMC Oral Health. 2024 May 24;24:605. doi: 10.1186/s12903-024-04358-8 (PMC11127407; doi:10.1186/s12903-024-04358-8)
Supplement: Supplementary file 1 — Supplementary Material 1 [file 12903_2024_4358_MOESM1_ESM.docx]

**ChatGPT-4 Answers (Texts in *italics* were generated by ChatGPT-4)**

**Q.1. What Is Dental Amalgam?**

*“Dental amalgam is a commonly used filling material in dentistry. It is a mixture of metals that includes silver, tin, copper, and mercury. The amalgam is prepared by mixing the metals together in a precise ratio, which results in a pliable material that can be easily placed into a prepared cavity in a tooth. Once the dental amalgam is placed in the tooth, it hardens and becomes a durable and long-lasting filling. Dental amalgam is used to fill cavities caused by tooth decay and can be used in both primary (baby) teeth and permanent teeth. Although dental amalgam has been used for over a century, there is ongoing debate about its safety due to the presence of mercury. However, many organizations including the American Dental Association (ADA), the U.S. Food and Drug Administration (FDA), and the World Health Organization (WHO) have concluded that dental amalgam is a safe and effective material for dental restorations.”*

**Q.2. Is Dental Amalgam Safe?**

*“The safety of dental amalgam has been a subject of ongoing debate and research for many years. Dental amalgam contains mercury, which is a toxic substance. However, the mercury in dental amalgam is bound in a stable compound that is not easily absorbed by the body, and studies have shown that the amount of mercury released by dental amalgam fillings is very low and within safe limits. Many organizations, including the American Dental Association, the U.S. Food and Drug Administration, and the World Health Organization, have concluded that dental amalgam is safe and effective for use in dental restorations. They have reviewed scientific evidence and conducted extensive research on the use of dental amalgam and have not found any evidence that it causes harm to patients. That being said, some people may have an allergy or sensitivity to one or more of the metals in dental amalgam, and they may experience an allergic reaction or other side effects as a result. Additionally, some patients may prefer to avoid dental amalgam due to concerns about mercury toxicity, and alternative dental filling materials are available. Ultimately, the decision to use dental amalgam or another type of dental filling material should be made in consultation with a dentist, based on the patient's individual needs and preferences.”*

**Q.3. Who Should Be Concerned About Dental Amalgam?**

***“****While dental amalgam is considered safe for most people, there are certain groups who may be more at risk of negative effects from exposure to mercury, and therefore may want to avoid or limit their exposure to dental amalgam. Pregnant women and young children are two groups that may be more susceptible to the potential harmful effects of mercury exposure. Pregnant women who are exposed to high levels of mercury may pass it to their developing fetuses, which can lead to developmental problems. Children, whose brains are still developing, may also be more sensitive to the effects of mercury exposure. People with certain health conditions, such as kidney problems or allergies to the metals in dental amalgam, may also want to avoid using this material. Additionally, individuals who have a large number of existing amalgam fillings may be at greater risk of mercury exposure, as the release of mercury from these fillings can increase with wear and tear over time. Overall, if you have concerns about dental amalgam and its potential effects on your health, it is important to discuss your options with a qualified dental professional, who can help you weigh the potential risks and benefits and determine the best course of action for your individual needs and circumstances.”*

**Q.4. Should Dental Amalgam Fillings Be Removed?**

***“****The decision to remove dental amalgam fillings should be made on an individual basis, in consultation with a qualified dental professional. While dental amalgam fillings do contain small amounts of mercury, scientific evidence suggests that the amount of mercury released by these fillings is very low and generally considered safe.* *Removing dental amalgam fillings can actually increase exposure to mercury, as the process of removal can release small amounts of mercury vapor and particles. Therefore, the American Dental Association and other organizations generally do not recommend the removal of sound amalgam fillings, or the replacement of amalgam fillings with other materials, solely for the purpose of reducing exposure to mercury.* *However, there are some situations in which the removal of dental amalgam fillings may be recommended. For example, if a filling is broken, worn, or decayed and needs to be replaced for dental health reasons, a patient may choose to replace it with a different type of filling material. Additionally, if a patient has a known allergy or sensitivity to one or more of the metals in dental amalgam, they may choose to have their existing fillings removed and replaced with an alternative material.* *Overall, the decision to remove dental amalgam fillings should be made in consultation with a qualified dental professional, based on the individual's specific dental and medical needs and circumstances.”*
