# Supplementary material for: Evaluating the accuracy of Chat Generative Pre-trained Transformer version 4 (ChatGPT-4) responses to United States Food and Drug Administration (FDA) frequently asked questions about dental amalgam
Source: BMC Oral Health. 2024 May 24;24:605. doi: 10.1186/s12903-024-04358-8 (PMC11127407; doi:10.1186/s12903-024-04358-8)
Supplement: Supplementary file 2 — Supplementary Material 2 [file 12903_2024_4358_MOESM2_ESM.docx]

**The Main Idea Analysis Of ChatGPT-4 Answers and FDA Guidance For The Same Questions (Texts in *italics* were generated by ChatGPT-4)**

**ChatGPT-4**

**Q.1. What Is Dental Amalgam?**

***A.1:*** *“Dental amalgam is a commonly used filling material in dentistry. It is a mixture of metals that includes silver, tin, copper, and mercury. The amalgam is prepared by mixing the metals together in a precise ratio, which results in a pliable material that can be easily placed into a prepared cavity in a tooth. Once the dental amalgam is placed in the tooth, it hardens and becomes a durable and long-lasting filling. Dental amalgam is used to fill cavities caused by tooth decay and can be used in both primary (baby) teeth and permanent teeth. Although dental amalgam has been used for over a century, there is ongoing debate about its safety due to the presence of mercury. However, many organizations including the American Dental Association (ADA), the U.S. Food and Drug Administration (FDA), and the World Health Organization (WHO) have concluded that dental amalgam is a safe and effective material for dental restorations.”*

**Q.1.1** **Please identify the central theme or message of this text.**

***A.1.1:*** *“ The central theme of the answer is the safety and effectiveness of dental amalgam as a filling material in dentistry. It highlights that despite being a mixture that includes mercury, dental amalgam has been deemed safe and effective for dental restorations by various reputable organizations such as the American Dental Association (ADA), the U.S. Food and Drug Administration (FDA), and the World Health Organization (WHO).”*

**Q.2. Is Dental Amalgam Safe?**

***A.2:****“The safety of dental amalgam has been a subject of ongoing debate and research for many years. Dental amalgam contains mercury, which is a toxic substance. However, the mercury in dental amalgam is bound in a stable compound that is not easily absorbed by the body, and studies have shown that the amount of mercury released by dental amalgam fillings is very low and within safe limits. Many organizations, including the American Dental Association, the U.S. Food and Drug Administration, and the World Health Organization, have concluded that dental amalgam is safe and effective for use in dental restorations. They have reviewed scientific evidence and conducted extensive research on the use of dental amalgam and have not found any evidence that it causes harm to patients. That being said, some people may have an allergy or sensitivity to one or more of the metals in dental amalgam, and they may experience an allergic reaction or other side effects as a result. Additionally, some patients may prefer to avoid dental amalgam due to concerns about mercury toxicity, and alternative dental filling materials are available. Ultimately, the decision to use dental amalgam or another type of dental filling material should be made in consultation with a dentist, based on the patient's individual needs and preferences.”*

**Q.2.2:** **Please identify the central theme or message of this text.**

***A.2.2:*** *“The central theme of the answer is the ongoing debate and research surrounding the safety of dental amalgam, particularly due to its mercury content. It discusses the conclusions drawn by various organizations such as the American Dental Association, the U.S. Food and Drug Administration, and the World Health Organization regarding the safety and effectiveness of dental amalgam. It emphasizes that while dental amalgam is generally considered safe for most patients, some individuals may have allergies or sensitivities to its components, and alternative filling materials are available. The text underscores the importance of making informed decisions about dental filling materials in consultation with a dentist, considering individual needs and preferences.”*

**Q.3. Who Should Be Concerned About Dental Amalgam?**

***A.3: “****While dental amalgam is considered safe for most people, there are certain groups who may be more at risk of negative effects from exposure to mercury, and therefore may want to avoid or limit their exposure to dental amalgam. Pregnant women and young children are two groups that may be more susceptible to the potential harmful effects of mercury exposure. Pregnant women who are exposed to high levels of mercury may pass it to their developing fetuses, which can lead to developmental problems. Children, whose brains are still developing, may also be more sensitive to the effects of mercury exposure. People with certain health conditions, such as kidney problems or allergies to the metals in dental amalgam, may also want to avoid using this material. Additionally, individuals who have a large number of existing amalgam fillings may be at greater risk of mercury exposure, as the release of mercury from these fillings can increase with wear and tear over time. Overall, if you have concerns about dental amalgam and its potential effects on your health, it is important to discuss your options with a qualified dental professional, who can help you weigh the potential risks and benefits and determine the best course of action for your individual needs and circumstances.”*

**Q.3.3:** **Please identify the central theme or message of this text.**

***A.3.3:*** *“The central theme of the answer is the consideration of certain groups who may be more vulnerable to the potential negative effects of mercury exposure from dental amalgam. It highlights pregnant women, young children, individuals with specific health conditions, and those with a large number of existing amalgam fillings as being at higher risk. The text emphasizes the importance of discussing concerns with a qualified dental professional to determine the best course of action based on individual circumstances and needs.”*

**Q.4. Should Dental Amalgam Fillings Be Removed?**

***A.4: “****The decision to remove dental amalgam fillings should be made on an individual basis, in consultation with a qualified dental professional. While dental amalgam fillings do contain small amounts of mercury, scientific evidence suggests that the amount of mercury released by these fillings is very low and generally considered safe.* *Removing dental amalgam fillings can actually increase exposure to mercury, as the process of removal can release small amounts of mercury vapor and particles. Therefore, the American Dental Association and other organizations generally do not recommend the removal of sound amalgam fillings, or the replacement of amalgam fillings with other materials, solely for the purpose of reducing exposure to mercury.* *However, there are some situations in which the removal of dental amalgam fillings may be recommended. For example, if a filling is broken, worn, or decayed and needs to be replaced for dental health reasons, a patient may choose to replace it with a different type of filling material. Additionally, if a patient has a known allergy or sensitivity to one or more of the metals in dental amalgam, they may choose to have their existing fillings removed and replaced with an alternative material.* *Overall, the decision to remove dental amalgam fillings should be made in consultation with a qualified dental professional, based on the individual's specific dental and medical needs and circumstances.”*

**Q.4.4:** **Please identify the central theme or message of this text.**

***A.4.4: “****The central theme of the answer is the importance of making informed decisions about the removal of dental amalgam fillings in consultation with a qualified dental professional. It discusses the low levels of mercury released by dental amalgam fillings and cautions against unnecessary removal solely for the purpose of reducing mercury exposure. The text emphasizes that while there are situations where removal may be recommended, such as for dental health reasons or due to allergies or sensitivities, the decision should be based on individual needs and circumstances and made in consultation with a dental professional.”*

**FDA Informations**

**Q.1. What Is Dental Amalgam?**

**A.1:** Dental amalgam, sometimes called a “silver-filling” due to its appearance, is a mixture of mercury, silver, copper, tin and zinc used to fill cavities in teeth. Dental amalgam is approximately half (50%) mercury, by weight.

**Q.1.1** **Please identify the central theme or message of this text.**

***A.1.1:*** *“The central theme of the answer is to provide a basic description of dental amalgam, outlining its composition and common name ("silver-filling") due to its appearance. It highlights the mixture of metals used in dental amalgam, including mercury, silver, copper, tin, and zinc, with a specific emphasis on the significant proportion of mercury in the mixture (approximately 50% by weight).”*

**Q.2. Is Dental Amalgam Safe?**

**A.2:** Dental amalgam fillings may release small amounts of mercury in the form of a vapor (gas), depending on the number and age of existing fillings, and actions such as tooth grinding and gum chewing. When a new amalgam filling is placed or an old filling is removed, patients and healthcare providers may experience a temporary increase in exposure to mercury vapor. While there are no known health risks associated with ingestion (swallowing) of small particles of dental amalgam, inhaling (breathing in) mercury vapors may be harmful in certain patients. In general, people with multiple dental amalgam fillings may have slightly higher mercury levels in their blood or urine, however, they usually remain at a level considered safe. Studies on people with dental amalgam do not show conclusive evidence that dental amalgam causes harmful health effects in the general population.

**Q.2.2:** **Please identify the central theme or message of this text.**

***A.2.2:*** *“The central theme of the answer is to discuss the potential release of mercury vapor from dental amalgam fillings and its implications for health. It highlights that while small amounts of mercury vapor may be released, particularly during placement or removal of fillings, there is no known health risk associated with ingesting small particles of dental amalgam. However, inhaling mercury vapors may pose a risk to certain individuals. The text emphasizes that studies have not shown conclusive evidence linking dental amalgam to harmful health effects in the general population, although individuals with multiple dental amalgam fillings may have slightly higher mercury levels in their blood or urine.”*

**Q.3. Who Should Be Concerned About Dental Amalgam?**

**A.3: “**Certain people, including women who are pregnant or who are planning to become pregnant, nursing mothers, children (especially those under the age of six), people with a known allergy to mercury, and people with neurological impairment or kidney dysfunction, may be more susceptible to the effects of exposure to mercury from dental amalgam and may be at greater risk for adverse health effects. Little information is known about the potential health effects of mercury vapor exposure from dental amalgam on these groups of people.

• Women who are pregnant or planning to become pregnant. Placement of new amalgam fillings in a pregnant mother may result in high, transient spikes of mercury exposure to the mother and fetus. Some studies have shown a relationship between the number of amalgam fillings a mother has and mercury levels in umbilical cord blood. Results from these studies did not identify any certain associations with harmful health effects; however, the data is very limited.

• Nursing mothers. The amount of mercury in breast milk is typically very low. Some studies have reported a relationship between the number of amalgam fillings a mother has and the amount of mercury in breast milk. The very few, limited studies that have been conducted to evaluate possible harm to infants and children exposed to dental amalgam mercury as a result of breast-feeding did not identify any definite associations with harmful health effects.

• Children, especially those under the age of six. Clinical studies in children ages six and older have not found a definite connection between the use of dental amalgam and harmful health effects. Studies on children under the age of six are very limited. The developing neurological systems of children may be especially sensitive to exposure to mercury vapor.

• People who are more sensitive to mercury or other components of dental amalgam. Some people may have an allergy or sensitivity to mercury or other components (e.g., silver, copper, tin or zinc) of dental amalgam and may report or experience health effects, including allergic reactions and oral ulcers, as well as more generalized symptoms, after placement of an amalgam filling.

• People with neurological impairment or kidney dysfunction. Studies show mercury is localized in certain tissues of the body including the brain and kidneys. Very limited clinical information is available about possible health effects of mercury vapor exposure on individuals who already have kidney and/or neurological impairments. Although a direct correlation between mercury from amalgam and possible harmful health effects has not been established, and there may be cumulative effects from exposure to mercury from other sources such as the environment or diet, some signs or symptoms of excessive mercury in the body may include:

• Mood disorders (for example, anxiety, depression, irritability)

• Sleep troubles or disturbances

• Fatigue (feeling tired)

• Memory troubles or disturbances

• Tremors (shaking)

• Difficulties with coordination

• Visual changes

• Changes in hearing

**Q.3.3:** **Please identify the central theme or message of this text.**

***A.3.3:****“The central theme of the answer is to raise awareness about certain groups of individuals who may be more vulnerable to the potential adverse health effects of mercury exposure from dental amalgam. It emphasizes the need for caution and consideration when it comes to the placement of amalgam fillings in pregnant or nursing women, young children, individuals with allergies or sensitivities to mercury or other components of amalgam, and those with neurological impairment or kidney dysfunction. The text underscores the importance of further research in understanding the potential health impacts of mercury vapor exposure, particularly on vulnerable populations, and highlights some possible signs or symptoms of excessive mercury exposure. Overall, the text aims to inform healthcare providers and patients about the potential risks associated with dental amalgam and the need for individualized assessment and decision-making.”*

**Q.4. Should Dental Amalgam Fillings Be Removed?**

**A.4:** If your filling is in good condition and your dentist or health care professional says there is no decay beneath the filling, removal of your amalgam filling is not recommended. This is because removing intact amalgam fillings may result in unnecessary loss of healthy tooth structure and potentially exposes you to a temporary increase in mercury vapor released during the removal process. Intact amalgam fillings in anyone, including those of greater risk such as pregnant or nursing mothers and children, should not be removed for the purpose of preventing any disease or health condition, unless considered medically necessary by a health care professional. If you have a health condition (especially sensitivity or allergy to mercury, or neurological or kidney disease), you should discuss the need for removal and replacement with your dentist or physician.

**Q.4.4:** **Please identify the central theme or message of this text.**

***A.4.4: “****The central theme of the answer is to emphasize that the removal of intact dental amalgam fillings is not recommended unless there is a specific dental or medical necessity determined by a healthcare professional. It highlights the potential risks associated with unnecessary removal, such as loss of healthy tooth structure and temporary exposure to increased mercury vapor during the removal process. The text stresses that even for individuals at greater risk, such as pregnant or nursing mothers and children, removal should only be considered if medically necessary. It underscores the importance of discussing the need for removal and replacement with a dentist or physician, particularly for individuals with specific health conditions or sensitivities.”*
