# Supplementary material for: Evaluating the accuracy of Chat Generative Pre-trained Transformer version 4 (ChatGPT-4) responses to United States Food and Drug Administration (FDA) frequently asked questions about dental amalgam
Source: BMC Oral Health. 2024 May 24;24:605. doi: 10.1186/s12903-024-04358-8 (PMC11127407; doi:10.1186/s12903-024-04358-8)
Supplement: Supplementary file 3 — Supplementary Material 3 [file 12903_2024_4358_MOESM3_ESM.docx]

**The Summary of Quality Analysis Chatgpt-4 Answers and FDA Guidance for The Same Questions.**

**a. Relevance**

a.1. Does the answer address the core of the question and avoid straying off topic?

a.2. Does the answer stay within the intended scope of the question?

**b. Accuracy**

b.1. Is the information factually correct and verified where possible (or evidence-supported)?

b.1.1. Can the information be supported by relevant references?

b.2. Are there any misleading or contradictory statements?

**c. Completeness**

c.1. Does the answer provide enough information to fully answer the question?

c.2. Are there any key points missing or left vague?

**d. Clarity**

d.1. Is the language clear, concise, and easy to understand?

d.2. Are there any jargon or complex terms that may hinder understanding?

| **Q1** | | **What Is Dental Amalgam?** | |
| --- | --- | --- | --- |
|  |  | **ChatGPT-4** | **FDA** |
| **Relevance** | ***a.1.*** | Yes, the answer effectively addresses the core of the question by providing a clear and concise explanation of what dental amalgam is. Therefore, it avoids straying off topic and provides relevant information regarding dental amalgam. | Yes, the answer provided succinctly addresses the question by defining dental amalgam as a mixture of specific metals used for filling cavities in teeth. |
|  | ***a.2.*** | Yes, the answer remains focused on addressing the core aspects of the question without deviating from its intended scope. It also briefly touches on the safety and effectiveness of dental amalgam, which is relevant to understanding its nature. | Yes, the answer stays within the intended scope of the question. It specifically discusses what dental amalgam is, its composition, and its purpose, which directly aligns with the question asked. |
| **Accuracy** | ***b.1.*** | The information provided in the answer appears to be factually correct and evidence-supported. | Yes, the information provided in the answer is factually correct and widely supported by evidence. Details are widely recognized in the field of dentistry and supported by scientific research and dental practice. |
|  | ***b.1.1.*** | Yes, the information provided in the answer can be supported by relevant references. | No. References should offer support for the factual accuracy of the information regarding dental amalgam as provided in the answer. |
|  | ***b.2.*** | The answer appears to be clear and free of any misleading or contradictory statements. The information provided is coherent and consistent throughout. | There are no misleading or contradictory statements in the answer. |
| **Completeness** | ***c.1.*** | The answer sufficiently addresses the question by providing a clear and concise explanation of what dental amalgam is and its role in dentistry. It gives a comprehensive overview of dental amalgam, including its composition, preparation, application, and the conclusions of various reputable organizations regarding its safety and effectiveness for dental restorations. | The answer adequately addresses the question by providing a comprehensive overview of dental amalgam. |
|  | ***c.2.*** | The answer provides a comprehensive overview of the definition of dental amalgam. | The answer provides a solid overview of dental amalgam. It could offer a more comprehensive understanding of dental amalgam and its role in dentistry. |
| **Clarity** | ***d.1.*** | The answer provides a straightforward explanation of what dental amalgam is, how it is prepared and used, and its safety and effectiveness according to various reputable organizations. The sentences are well-structured, and technical terms are explained in a way that is accessible to a general audience. | The language effectively communicates the intended information without unnecessary complexity. |
|  | ***d.2.*** | Technical terms such as "dental amalgam," "filling material," "mixture of metals," and "cavity in a tooth" are commonly understood in the context of dentistry and are explained in a way that is easy to grasp. Therefore, the answer effectively avoids using jargon or complex terms that may hinder understanding. | The answer is easily comprehensible to a general audience without the use of overly complex terminology. |
| **Q2** | | **Is Dental Amalgam Safe?** | |
|  |  | **ChatGPT-4** | **FDA** |
| **Relevance** | ***a.1.*** | The response remains focused on the safety of dental amalgam and relevant considerations without straying off topic. | Yes, the answer appropriately addresses the core of the question regarding the safety of dental amalgam. The response stays focused on the safety aspect of dental amalgam without straying off-topic. |
|  | ***a.2.*** | The response remains focused on addressing the concerns surrounding the safety of dental amalgam and does not stray from this topic. | Yes, the answer remains within the intended scope of the question, which is about the safety of dental amalgam. Therefore, it effectively stays within the intended scope of the question without veering off-topic. |
| **Accuracy** | ***b.1.*** | Yes, the information provided in the answer is factually correct and supported by evidence where possible. | The information provided appears to be factual and evidence-supported based on current knowledge, but it's always recommended to consult with dental and medical professionals for personalized advice and updates on health-related matters. |
|  | ***b.1.1.*** | Yes, the information provided in the answer can be supported by relevant references. | The references provide scientific evidence and research findings supporting various aspects of the safety of dental amalgam, including mercury vapor release, health risks, mercury levels in the body, and general safety considerations. |
|  | ***b.2.*** | While the answer provides a comprehensive overview of the current understanding of dental amalgam safety, readers should be aware of the ongoing debate and consult multiple sources for a balanced perspective. | Upon reviewing the provided information, there don't appear to be any glaringly misleading or contradictory statements. |
| **Completeness** | ***c.1.*** | While it's a complex topic with ongoing debate, the answer gives a comprehensive overview of the current understanding of dental amalgam safety, enabling readers to make informed decisions. | While further details could always be provided for a more comprehensive understanding, the answer sufficiently addresses the core aspects of the question regarding the safety of dental amalgam. |
|  | ***c.2.*** | By addressing the key points about Long-term Effects, Environmental Impact, Individual Risk Factors and Alternative Materials the answer would provide a more comprehensive understanding of the safety considerations and factors involved in the use of dental amalgam. | The answer provides a comprehensive overview of the safety considerations surrounding dental amalgam, |
| **Clarity** | ***d.1.*** | Yes, the language used in the answer is clear, concise, and easy to understand. | The answer effectively communicates key points without excessive technical jargon, enhancing clarity and comprehension. |
|  | ***d.2.*** | The answer generally avoids jargon and complex terms, making it accessible to a wide audience. | The answer primarily avoids jargon and complex terms, making it generally accessible. |
| **Q3** | | **Who Should Be Concerned About Dental Amalgam?** | |
|  |  | **ChatGPT-4** | **FDA** |
| **Relevance** | ***a.1.*** | Yes, the answer effectively addresses the core of the question by discussing who should be concerned about dental amalgam and why. | Yes, the answer addresses the core of the question by identifying specific groups of people who should be concerned about dental amalgam due to potential health risks associated with mercury exposure. |
|  | ***a.2.*** | Yes, the answer stays within the intended scope of the question. | Yes, the answer stays within the intended scope of the question, which is about identifying who should be concerned about dental amalgam. Therefore, it effectively addresses the question without straying off-topic. |
| **Accuracy** | ***b.1.*** | Yes, the information provided in the answer is factually correct and supported by evidence. the answer presents factual and evidence-supported information regarding concerns about dental amalgam. | The information presented seems to be accurate and supported by evidence to the best of current knowledge. |
|  | ***b.1.1.*** | The references support the information provided in the answer and offer additional resources for further reading and verification. | The references could include scientific papers, government health agency reports, or reputable medical organizations' guidelines on dental amalgam and its potential health effects. Incorporating such references would strengthen the evidence base and allow readers to explore the topic further. |
|  | ***b.2.*** | No, there are no misleading or contradictory statements in the provided response. | The information seems accurate and informative, but readers should consult multiple sources for a comprehensive understanding of the subject. |
| **Completeness** | ***c.1.*** | Yes, the answer provides sufficient information to fully address the question. | The answer sufficiently covers the topic and provides relevant details to fully answer the question. |
|  | ***c.2.*** | While the answer provides comprehensive information about groups who may be more susceptible to the potential harmful effects of mercury exposure from dental amalgam, it could further elaborate on the potential risks associated with mercury exposure for these groups. For example, it could mention specific developmental problems that may occur in fetuses exposed to high levels of mercury, as well as the neurological impacts that mercury exposure can have on children. Additionally, it could provide more details on the health conditions that may increase susceptibility to mercury exposure and explain how amalgam fillings release mercury over time, contributing to potential risks. | While the answer provides a thorough overview of who should be concerned about dental amalgam, there are a few key points that could be further emphasized or clarified. “Risk Assessment for Individuals with Allergies or Sensitivities”, “Potential Long-Term Health Effects”, “Cumulative Mercury Exposure”, “Preventive Measures”. By addressing these points, the answer could provide a more comprehensive understanding of the concerns surrounding dental amalgam and its potential impact on various populations. |
| **Clarity** | ***d.1.*** | Yes, the language used in the answer is clear, concise, and easy to understand. | The language used enhances the clarity and accessibility of the information provided. |
|  | ***d.2.*** | the language used is clear and accessible, but providing additional context for terms like "amalgam fillings" and elaborating on certain concepts could improve understanding. | The answer generally avoids using jargon or complex terms that might hinder understanding. |
| **Q4** | | **Should Dental Amalgam Fillings Be Removed?** | |
|  |  | **ChatGPT-4** | **FDA** |
| **Relevance** | ***a.1.*** | Yes, the answer effectively addresses the core of the question by providing a thorough explanation of the considerations surrounding the removal of dental amalgam fillings. It provides a comprehensive and balanced perspective on the issue. | The answer stays focused on the topic of dental amalgam fillings and their removal, without straying off into unrelated areas. Overall, it effectively addresses the core concerns related to the question. |
|  | ***a.2.*** | Yes, the answer stays within the intended scope of the question. | Yes, the answer stays within the intended scope of the question. It remains squarely within the scope of the question. |
| **Accuracy** | ***b.1.*** | The response appears to be well-supported by credible sources and presents information that aligns with current understanding in the field of dentistry. | The information presented in the answer is both factually correct and evidence-supported. |
|  | ***b.1.1.*** | Yes, the information provided in the answer can be supported by relevant references. | The references support the information provided in the answer regarding the considerations for removal of dental amalgam fillings and the importance of professional guidance in making such decisions. |
|  | ***b.2.*** | The information provided is consistent and does not contain any misleading or contradictory statements. | The information provided is clear, consistent, and aligns with established guidelines from reputable organizations such as the American Dental Association, the U.S. Food and Drug Administration, the Centers for Disease Control and Prevention, and the World Health Organization. |
| **Completeness** | ***c.1.*** | The answer offers a well-rounded perspective on the topic, allowing readers to make informed decisions about dental amalgam fillings and their potential removal. | The answer offers comprehensive guidance on the topic, providing the necessary information for individuals to make informed decisions about the removal of dental amalgam fillings. |
|  | ***c.2.*** | The answer covers the main points related to the removal of dental amalgam fillings, but there are a few additional aspects that could be considered to provide a more comprehensive understanding: Environmental Impact, Alternatives to Amalgam, Long-Term Health Effects, Cost Considerations. | While the answer provides comprehensive guidance on the topic of whether dental amalgam fillings should be removed, there are a few additional key points that could be addressed or expanded upon: Long-term considerations, Alternatives to amalgam fillings, Potential benefits of removal, Risk mitigation during removal. |
| **Clarity** | ***d.1.*** | The language used ensures accessibility for readers of various backgrounds and levels of familiarity with the topic. | The clarity and simplicity of the language contribute to the effectiveness of the answer in addressing the question. |
|  | ***d.2.*** | While the answer generally uses accessible language, providing brief explanations for terms that may not be universally understood could enhance comprehension for all readers. | The answer does not contain any jargon or complex terms that may hinder understanding. Readers are unlikely to encounter any barriers to understanding due to jargon or complex terminology in the provided answer. |
