# Supplementary material for: Evaluating the accuracy of Chat Generative Pre-trained Transformer version 4 (ChatGPT-4) responses to United States Food and Drug Administration (FDA) frequently asked questions about dental amalgam
Source: BMC Oral Health. 2024 May 24;24:605. doi: 10.1186/s12903-024-04358-8 (PMC11127407; doi:10.1186/s12903-024-04358-8)
Supplement: Supplementary file 4 — Supplementary Material 4 [file 12903_2024_4358_MOESM4_ESM.docx]

**The Summary of Common Themes, Concepts, or Information in Responses From Chatgpt-4 and FDA Guidance to the Same Questions (Texts in *italics* were generated by ChatGPT-4)**

**A. Comparison of the Answers to the First Question**

**Text 1 (***ChatGPT-4***)**

*“Dental amalgam is a commonly used filling material in dentistry. It is a mixture of metals that includes silver, tin, copper, and mercury. The amalgam is prepared by mixing the metals together in a precise ratio, which results in a pliable material that can be easily placed into a prepared cavity in a tooth. Once the dental amalgam is placed in the tooth, it hardens and becomes a durable and long-lasting filling. Dental amalgam is used to fill cavities caused by tooth decay and can be used in both primary (baby) teeth and permanent teeth. Although dental amalgam has been used for over a century, there is ongoing debate about its safety due to the presence of mercury. However, many organizations including the American Dental Association (ADA), the U.S. Food and Drug Administration (FDA), and the World Health Organization (WHO) have concluded that dental amalgam is a safe and effective material for dental restorations.”*

**Text 2** *(FDA)*

Dental amalgam, sometimes called a “silver-filling” due to its appearance, is a mixture of mercury, silver, copper, tin and zinc used to fill cavities in teeth. Dental amalgam is approximately half (50%) mercury, by weight.

Please Identify overlapping themes, concepts, or information presented in both texts.

**ChatGPT Response;**

*“Both answers discuss dental amalgam, a common filling material used in dentistry, composed of various metals including mercury, silver, tin, and copper. They both mention the process of preparing and placing dental amalgam into tooth cavities. Additionally, both answers touch upon the debate surrounding the safety and effectiveness of dental amalgam, with ChatGPT-4 highlighting the endorsements from organizations such as the American Dental Association (ADA), the U.S. Food and Drug Administration (FDA), and the World Health Organization (WHO), while FDA emphasizes the mercury content in dental amalgam.”*

**B. Comparison of the Answers to the Second Question**

**Text 1 (***ChatGPT-4***)**

*“The safety of dental amalgam has been a subject of ongoing debate and research for many years. Dental amalgam contains mercury, which is a toxic substance. However, the mercury in dental amalgam is bound in a stable compound that is not easily absorbed by the body, and studies have shown that the amount of mercury released by dental amalgam fillings is very low and within safe limits. Many organizations, including the American Dental Association, the U.S. Food and Drug Administration, and the World Health Organization, have concluded that dental amalgam is safe and effective for use in dental restorations. They have reviewed scientific evidence and conducted extensive research on the use of dental amalgam and have not found any evidence that it causes harm to patients. That being said, some people may have an allergy or sensitivity to one or more of the metals in dental amalgam, and they may experience an allergic reaction or other side effects as a result. Additionally, some patients may prefer to avoid dental amalgam due to concerns about mercury toxicity, and alternative dental filling materials are available. Ultimately, the decision to use dental amalgam or another type of dental filling material should be made in consultation with a dentist, based on the patient's individual needs and preferences.”*

**Text 2** *(FDA)*

Dental amalgam fillings may release small amounts of mercury in the form of a vapor (gas), depending on the number and age of existing fillings, and actions such as tooth grinding and gum chewing. When a new amalgam filling is placed or an old filling is removed, patients and healthcare providers may experience a temporary increase in exposure to mercury vapor. While there are no known health risks associated with ingestion (swallowing) of small particles of dental amalgam, inhaling (breathing in) mercury vapors may be harmful in certain patients. In general, people with multiple dental amalgam fillings may have slightly higher mercury levels in their blood or urine, however, they usually remain at a level considered safe. Studies on people with dental amalgam do not show conclusive evidence that dental amalgam causes harmful health effects in the general population.

Please Identify overlapping themes, concepts, or information presented in both texts.

**ChatGPT-4 Response;**

**“***Both answers discuss the safety concerns regarding dental amalgam, particularly related to the presence of mercury and the potential for its release in vapor form. They also both mention the assessments conducted by various organizations, including the American Dental Association, the U.S. Food and Drug Administration, and the World Health Organization, regarding the safety of dental amalgam. Additionally, they both acknowledge the possibility of allergic reactions or sensitivities to the metals in dental amalgam, as well as the existence of alternative filling materials. Both answers emphasize the importance of informed decision-making in consultation with a dentist based on individual needs and preferences.”*

**C. Comparison of the Answers to the Third Question**

**Text 1 (***ChatGPT-4***)**

*“While dental amalgam is considered safe for most people, there are certain groups who may be more at risk of negative effects from exposure to mercury, and therefore may want to avoid or limit their exposure to dental amalgam. Pregnant women and young children are two groups that may be more susceptible to the potential harmful effects of mercury exposure. Pregnant women who are exposed to high levels of mercury may pass it to their developing fetuses, which can lead to developmental problems. Children, whose brains are still developing, may also be more sensitive to the effects of mercury exposure. People with certain health conditions, such as kidney problems or allergies to the metals in dental amalgam, may also want to avoid using this material. Additionally, individuals who have a large number of existing amalgam fillings may be at greater risk of mercury exposure, as the release of mercury from these fillings can increase with wear and tear over time. Overall, if you have concerns about dental amalgam and its potential effects on your health, it is important to discuss your options with a qualified dental professional, who can help you weigh the potential risks and benefits and determine the best course of action for your individual needs and circumstances.”*

**Text 2** *(FDA)*

Certain people, including women who are pregnant or who are planning to become pregnant, nursing mothers, children (especially those under the age of six), people with a known allergy to mercury, and people with neurological impairment or kidney dysfunction, may be more susceptible to the effects of exposure to mercury from dental amalgam and may be at greater risk for adverse health effects. Little information is known about the potential health effects of mercury vapor exposure from dental amalgam on these groups of people.

• Women who are pregnant or planning to become pregnant. Placement of new amalgam fillings in a pregnant mother may result in high, transient spikes of mercury exposure to the mother and fetus. Some studies have shown a relationship between the number of amalgam fillings a mother has and mercury levels in umbilical cord blood. Results from these studies did not identify any certain associations with harmful health effects; however, the data is very limited.

• Nursing mothers. The amount of mercury in breast milk is typically very low. Some studies have reported a relationship between the number of amalgam fillings a mother has and the amount of mercury in breast milk. The very few, limited studies that have been conducted to evaluate possible harm to infants and children exposed to dental amalgam mercury as a result of breast-feeding did not identify any definite associations with harmful health effects.

• Children, especially those under the age of six. Clinical studies in children ages six and older have not found a definite connection between the use of dental amalgam and harmful health effects. Studies on children under the age of six are very limited. The developing neurological systems of children may be especially sensitive to exposure to mercury vapor.

• People who are more sensitive to mercury or other components of dental amalgam. Some people may have an allergy or sensitivity to mercury or other components (e.g., silver, copper, tin or zinc) of dental amalgam and may report or experience health effects, including allergic reactions and oral ulcers, as well as more generalized symptoms, after placement of an amalgam filling.

• People with neurological impairment or kidney dysfunction. Studies show mercury is localized in certain tissues of the body including the brain and kidneys. Very limited clinical information is available about possible health effects of mercury vapor exposure on individuals who already have kidney and/or neurological impairments. Although a direct correlation between mercury from amalgam and possible harmful health effects has not been established, and there may be cumulative effects from exposure to mercury from other sources such as the environment or diet, some signs or symptoms of excessive mercury in the body may include:

• Mood disorders (for example, anxiety, depression, irritability)

• Sleep troubles or disturbances

• Fatigue (feeling tired)

• Memory troubles or disturbances

• Tremors (shaking)

• Difficulties with coordination

• Visual changes

• Changes in hearing

Please Identify overlapping themes, concepts, or information presented in both texts.

**ChatGPT-4 Response;**

**“***Both answers address the potential risks associated with exposure to mercury from dental amalgam, particularly for certain vulnerable groups such as pregnant women, young children, nursing mothers, and individuals with specific health conditions like allergies, neurological impairment, or kidney dysfunction. They both emphasize the importance of considering individual circumstances and discussing concerns with a qualified dental professional. Additionally, they both acknowledge the limited data available on the health effects of mercury exposure from dental amalgam in certain populations, such as pregnant women and young children, indicating the need for further research in this area.”*

**D. Comparison of the Answers to the Forth Question**

**Text 1 (***ChatGPT-4***)**

*“The decision to remove dental amalgam fillings should be made on an individual basis, in consultation with a qualified dental professional. While dental amalgam fillings do contain small amounts of mercury, scientific evidence suggests that the amount of mercury released by these fillings is very low and generally considered safe. Removing dental amalgam fillings can actually increase exposure to mercury, as the process of removal can release small amounts of mercury vapor and particles. Therefore, the American Dental Association and other organizations generally do not recommend the removal of sound amalgam fillings, or the replacement of amalgam fillings with other materials, solely for the purpose of reducing exposure to mercury. However, there are some situations in which the removal of dental amalgam fillings may be recommended. For example, if a filling is broken, worn, or decayed and needs to be replaced for dental health reasons, a patient may choose to replace it with a different type of filling material. Additionally, if a patient has a known allergy or sensitivity to one or more of the metals in dental amalgam, they may choose to have their existing fillings removed and replaced with an alternative material. Overall, the decision to remove dental amalgam fillings should be made in consultation with a qualified dental professional, based on the individual's specific dental and medical needs and circumstances.”*

**Text 2** *(FDA)*

If your filling is in good condition and your dentist or health care professional says there is no decay beneath the filling, removal of your amalgam filling is not recommended. This is because removing intact amalgam fillings may result in unnecessary loss of healthy tooth structure and potentially exposes you to a temporary increase in mercury vapor released during the removal process. Intact amalgam fillings in anyone, including those of greater risk such as pregnant or nursing mothers and children, should not be removed for the purpose of preventing any disease or health condition, unless considered medically necessary by a health care professional. If you have a health condition (especially sensitivity or allergy to mercury, or neurological or kidney disease), you should discuss the need for removal and replacement with your dentist or physician.

Please Identify overlapping themes, concepts, or information presented in both texts.

**ChatGPT-4 Response;**

*“Both answers emphasize the importance of individualized decision-making in consultation with a qualified dental professional regarding the removal of dental amalgam fillings. They both highlight that intact dental amalgam fillings, especially those in good condition without decay beneath them, generally do not need to be removed solely for the purpose of reducing exposure to mercury. Additionally, they both caution against unnecessary removal of amalgam fillings, as it may lead to loss of healthy tooth structure and temporary increase in mercury vapor exposure during the removal process. They both stress the need for considering medical necessity and specific health conditions when determining whether removal and replacement of amalgam fillings are warranted. Overall, both answers advocate for a careful assessment of individual circumstances and dental health needs before making decisions about dental amalgam removal.”*
